# Cellular response of limbal epithelial cells on electrospun poly-ε-caprolactone nanofibrous scaffolds for ocular surface bioengineering: a preliminary in vitro study

**Published:** 2011-11-12

**Authors:** Shweta Sharma, Sujata Mohanty, Deepika Gupta, Manjeet Jassal, Ashwini K. Agrawal, Radhika Tandon

**Affiliations:** 1 Department of Ophthalmology, Dr. Rajendra Prasad Centre for Ophthalmic Sciences, All India Institute of Medical Sciences, New Delhi, India; 2 Stem Cell Facility, All India Institute of Medical Sciences, New Delhi, India; 3 SMITA Research Labs, Department of Textile Technology, Indian Institute of Technology, Hauz Khas, New Delhi, India

## Abstract

**Purpose:**

The aim of this study was to develop a synthetic stromal substrate for limbal epithelial cell (LEC) expansion that can serve as a potential alternative substrate to replace human amniotic membrane (HAM).

**Methods:**

Nanofibers were fabricated using 10% poly-ε-caprolactone (PCL) solution dissolved in trifluoroethanol (TFE) via an electrospinning process. Nanofibers were characterized for surface morphology, wetting ability, pore size, mechanical strength, and optical transparency using scanning electron microscopy (SEM), contact angle measurement, microtensile tester, and UV-Vis spectrophotometer, respectively. The human corneal epithelial (HCE-T) cell line was used to evaluate the biocompatibility of nanofibers based on their phenotypic profile, viability, proliferation, and attachment ability. Subsequently, human LECs were cultivated on biocompatible nanofibers for two weeks and their proliferation capability analyzed using MTT ((3-(4,5-Dimethylthiazol-2-yl)-2,5-diphenyltetrazolium bromide, a yellow tetrazole)) proliferation assay. Immunofluorescent (IF) staining and reverse transcriptase polymerase chain reaction (RT–PCR) were performed to check the molecular marker expression; SEM was used to study the morphology.

**Results:**

The average fiber diameter of PCL was 132±42 nm. Pore size varied from 0.2 to 4 microns with a porosity of 85%. The tensile strength of the PCL membrane was 1.74±0.18 MPa (Mega Pascal); strain was 30.08±2.66%. The water contact angle was 90°. Biocompatibility results indicated that the polymer surface was highly biocompatible, as HCE-T cells could favorably attach and proliferate on the polymer surface. SEM figures showed that the corneal epithelium was firmly anchored to the polymer surface via a continuous cell sheet and was able to retain a normal corneal phenotype. MTT assay confirmed that cells were metabolically active on nanofibers (p˂0.05) and gradually increased in their number for up to two weeks. IF and RT–PCR results revealed no change in the expression profile of LECs grown on nanofibers when compared to those grown on glass coverslips and human amniotic membrane (HAM). Confocal microscopy illustrated that cells infiltrated the nanofibers and successfully formed a three-dimensional (3D) corneal epithelium, which was viable for two weeks.

**Conclusions:**

Electrospun nanofibers provide not only a milieu supporting LEC expansion, but also serve as a useful alternative carrier for ocular surface tissue engineering and could be used as an alternative substrate to HAM.

## Introduction

Dysfunction or loss of limbal epithelial stem cells produces varying degrees of limbal stem cell deficiency (LSCD), which may lead to decreased vision, ocular discomfort, pain, and an unstable ocular surface [1]. Transplantation of ex-vivo expanded limbal epithelial cells (LECs) has been found as a promising procedure to treat corneas manifesting LSCD [1-4]. Advances in tissue engineering have allowed for the use of different substrates as a scaffold for expansion of LECs [1-10]. Human amniotic membrane (HAM) is the most widely used substrate for construction of damaged ocular surface, and has been considered as a gold standard scaffold for LEC expansion [2,4,11-13]. However, HAM is an allogenic biologic material and is associated with certain disadvantages, including disease transmission (human immunodeficiency virus [HIV], Hepatitis B and C), contamination, limited tissue availability, shelf life, specific storage conditions (−86 °C), and biologic variability between donor tissues [14,15].

The use of synthetic stromal substitute can therefore overcome these limitations for ocular surface reconstruction. Recently, many alternative materials have been used for culturing LECs, many of which are under preclinical and clinical trials [5-10,16-18]. The main objective of this study is to fabricate a 3D, biocompatible scaffold that should be biomimetic for LECs and should work as a natural extracellular matrix (ECM). Here, 3D porous scaffolds were produced by electrospinning a poly-ε-caprolactone (PCL) solution and applying high voltage between the polymer solution and a collector. During electrospinning, as the polymer droplet flows from the needle tip, under the influence of high voltage, it experiences excessive stretching and thinning and draws into very fine fibers, each with a diameter of a few hundreds of nanometers. These nanofibers assemble into 3D patterns and closely mimic the ECM environment of the tissue, which is required for successful tissue engineering applications. Scaffolds thus produced possess desirable properties such as high porosity, high surface to volume ratio, and ease of handling [19-20].

PCL is a degradable aliphatic ester that the USA Food and Drug Administration (FDA) has approved for human clinical use. In ophthalmic applications, PCL has already been explored as a drug delivery agent and as a carrier to cultivate retinal and conjunctival progenitor cells due to its in-vivo biocompatibility and the fact that it does not induce any immunological reaction after degradation [21-23]. Extensive research has been conducted on PCL due to its advantages such as biocompatibility, low cost, ease of use with controlled pore size and shape, and appropriate mechanical strength [21,25]. However, its prospective use as scaffold material for LEC expansion has never been explored, to the best of our knowledge.

The present work aims to conduct a biocompatibility assessment of PCL nanofibers using an established human corneal epithelial cell line (HCE-T) and to complete a feasibility study for developing synthetic ocular surface reconstruction over these PCL scaffolds.

## Methods

### Preparation of PCL nanofibrous scaffolds

PCL solution (10% w/v) was made by dissolving PCL pellets in trifluoroethanol (TFE). The clear solution was electrospun using an electrospinning setup consisting of a dual polarity, high-voltage DC power supply unit (Gamma High Voltage Research, Ormond Beach, FL), a syringe pump (KDS 100, KD Scientific, Holliston, MA), syringe (Dispovan, Faridabad, India), and a needle (24 G) with blunted tip. The positive terminal of the high-voltage supply was connected to the needle tip while the negative terminal was connected to a metallic collector plate; a voltage of 15 kV was maintained between them. Electrospun fibers were collected on coverslips kept over the metallic collector plate. Flow rate was maintained at 0.5 ml/h and needle tip to collector distance was maintained at 13 cm. All chemicals were purchased from Sigma Aldrich (St. Louis, MO).

### Physical characterization of PCL nanofibrous scaffolds

#### Surface morphology of nanofibers

The surface morphology of nanofibers was studied using scanning electron microscopy (SEM; 200F; Quanta, Eindhoven, The Netherlands) at an accelerating voltage of 20 kV. Fiber diameter was analyzed using image analysis software (Image J, National Institutes of Health, Bethesda, MD).

#### Contact angle measurement and optical transparency

Hydrophobicity of scaffolds was measured using water contact angle measurement. A water droplet (30 micron) was placed on the membranes using a microsyringe and an image was taken with a digital camera. Finally, the contact angle was calculated using image analysis software. The test was performed for six samples and an average value was quoted.

PCL membranes deposited on the glass coverslip were kept in phosphate buffer saline (PBS) for 2 h, 24 h, and 48 h for wetting. Dry and wet PCL samples dipped in PBS for 48 h were used for an optical transparency test and were compared with wet HAM supported on a glass coverslip. The glass coverslip was used as a control. Percentage transmittance (%T) was measured using UV-VIS spectrophotometer (Model Lambda 35; Perkin Elmer, Singapore) in the visible range from 400 nm to 700 nm wavelength. Visual assessment of optical transparency of dry PCL, wet PCL, and wet HAM was also completed by keeping these membranes on a printed text and taking photographs using a digital camera.

### Tensile strength measurement

The tensile properties of membrane were measured using an Instron 5848 Microtester (High Wycombe, UK), at a cross head speed of 5 mm/min and load cell of 10 Newton capacity. Rectangular specimens with width of 10 mm, length of 30 mm, and thickness of 30 µm were used for the studies. Six specimens were tested and the average value was reported.

### Pore size and porosity

Pore size distribution of the scaffold was measured by capillary flow porometer CFP-1100-AEXLH (PMI Inc., Ithaca, NY) using the wet-up/dry-up method. The analysis was performed using Capwin software. Porosity of the scaffold was calculated using the method used by Wei et al. [26]. The bulk density of PCL was calculated as 1.146 g/cm^3^. Thickness of PCL membranes was measured by micrometer. Apparent density and porosity were calculated using the equations described below:

$${\text{Apparent density (g/cm}}^{3}\text{) = }\frac{\text{Mass of nanofiber membrane (g)}}{\text{Membrane thickness (cm) }\times \text{ area}}$$

$$\text{Porosity (\%) = (1 - }\frac{{\text{Apparent density (g/cm}}^{3}\text{)}}{\text{Bulk density of membranes}}\text{) }\times \text{ 100}$$

### Pre-conditioning of nanofibrous scaffolds

The scaffolds were washed with PBS containing antibiotics, and then irradiated using UV light for 3 h. Thereafter, the scaffolds were incubated with a culture medium at 37 °C overnight (O/N). Finally, the culture medium was discarded and the scaffolds were further used for cell culture experiments.

### Biocompatibility assessment of nanofibers

The HCE-T cell line (Riken cell bank, Tsukuba, Japan) was used to assess the biocompatibility of nanofibers. A total of 2.6×10^4^ cells/cm^2^ were seeded on nanofibers and glass coverslips, which served as a control in this study. Cultures were maintained in Dulbecco’s Modified Eagle Medium (DMEM), supplemented with 5% Fetal Bovine Serum (FBS) and 100 U/ml penicillin/streptomycin, 5 µg/ml insulin, and 10 ng/ml hEGF. Cultures were incubated at 37 °C. At the end of the incubation, cultures were checked for viability, morphology, proliferation, and attachment ability. All cell culture reagents were purchased from Sigma Aldrich (St. Louis, MO).

### Viability staining for cytotoxicity analysis

Cultures were subjected to viability staining using a Live-Dead cell staining kit (Biovision Research, Mountain View, CA) according to the manufacture’s protocol. Cultures were washed with PBS and stained with 1 mM Live-Dye^TM^ and 2.5 mg/ml propidium iodide (PI) for 15 min at 37 °C. After incubation, cultures were viewed under a fluorescent microscope (Nikon, Toyoko Japan). The dyes had an excitation of 488 nm and an emission at 518 nm and 615 nm for live dye and PI, respectively. A total of 500 cells were counted in five fields; the percentage of viable cells was calculated.

### SEM for morphology and attachment evaluation

The cultures were incubated at 37 °C for 6 h, 1, 3, 5, and 7 days. Cultures were fixed with 2.5% gluteraldehyde and were further dehydrated using sequential immersion in alcohol series, followed by dehydration with 1,1,1,3,3,3-hexamethyldisilazane (HMDS; Merck, Whitehouse Station, NJ). The samples were sputter-coated with gold and observed under SEM.

### Cultivation of LECs over nanofibers

A total of 30 human limbal tissue specimens were obtained from discarded donor sclerocorneal rims after keratoplasty. Mean donor age was 36.73±14.26 (range 17–59) years. The average time interval from tissue retrieval to culture was 1.73±1.0 (range 1–4) days. The LEC culture was initiated using the explant culture method. The explant culture was performed according to our previously reported method [27].

Briefly, the harvested limbal tissue pieces were placed on the surface of preconditioned nanofibers and left for 10 min. After 10 min, growth medium consisted of DMEM/F12 nutrient mixture (3:1) supplemented with FBS (10%), insulin (5 µg/ml), hydrocortisone (0.5 µg/ml), glutamine (2 mmol/l), hEGF (20 ng/ml), and penicillin. Streptomycin (100 U/ml) was added and incubated at 37 °C with 5% CO_2_. The cultures were maintained for 14 days and the medium was changed twice a week. All cell culture reagents were purchased from Sigma-Aldrich.

### Viability and proliferation staining

The viability staining of LECs cultured on nanofibers was performed using a Live-Dead cell staining kit (Biovision Research) according to the above-mentioned protocol. The samples were also viewed under confocal microscope (Nikon) for imaging 3D construct formation.

Proliferation and growth kinetics of LECs was measured colorimetrically with thiazolyl blue tetrazolium blue (MTT; Sigma-Aldrich) assay using standard protocol. Briefly, 2.6×10^4^ cells/cm^2^ cells were seeded on PCL nanofibers, HAM and glass coverslip. Cultures were maintained for 3 h, 7, 10, and 14 days, and subsequently processed for MTT assay by adding 5 mg/ml of MTT solution and being incubated for 3 h. Soluble purple color formazan crystals that formed were dissolved in dimethyl sulfoxide (DMSO). Absorbance was read at 490 nm using an ELISA reader (BioTek, VT, USA). All experiments were performed in triplicate and repeated three times.

### Morphological analysis

The morphology of LECs cultured for two weeks was analyzed using SEM according to the above-mentioned protocol.

### Molecular characterization

#### Immunofluorescence (IF)

Cultures were stained with mouse monoclonal antibody K3/12 (clone AE5; Chemicon, Temecula, CA), ABCG2 (clone 5D3; BD Pharmingen, San diego,  CA), and Integrin β1 (Clone LM534; Chemicon) at a dilution of 1:100. Cultures were fixed with cold methanol and acetone (3:1; for K3/12) for 5 min and 2% paraformaldehyde (for Integrin β1 at the end of the staining). Non-specific sites were blocked using 2% BSA (Jakson ImmunoResearch Lab, West Grove, PA), followed by incubation with primary antibodies overnight at 4 °C. The cells were then incubated with secondary FITC rat anti mouse IgG1 Ab (1:500; BD PharMingen) for 1 h and counterstained with DAPI (1:1000; Invitrogen, Carlsbad, CA). Finally, the cells were mounted with an antifade reagent (FluoroGuard; Bio Rad Laboratories, Hercules, CA) and observed under fluorescent microscope.

#### RT PCR

RNA was extracted using a Trizol reagent (Invitrogen) according to the manufacturer’s protocol. cDNA was synthesized using a moloney murine leukemia virus reverse transcriptase enzyme (Promega, Madison, WI). Glyceraldehyde-3-phosphate dehydrogenase (*GAPDH*) was used as an internal control. The expression of stem cell Cytokeratin (K) 19 and ATP-binding cassette sub-family G member 2 (*ABCG2*) and differentiation markers (*K3* and *K12*) was checked using routine PCR (Table 1).

**Table 1:** Primer Sequences used for RT PCR.

**Gene**	**Sense Primer**	**Antisense Primer**	**Product Size (bp)**
*K12*	ACATGAAGAAGAACCACGAGGATG	TCTGCTCAGCGATGGTTTCA	150
*K19*	TGAGGTCATGGCCGAGCAGAAC	CATGAGCCGCTGGTACTCCTGA	331
*ABCG2*	AGTTCCATGGCACTGGCCATA	TCAGGTAGGCAATTGTGAGG	379
*K3*	GGCAGAGATCGAGGGTGTC	GTCATCCTTCGCCTGCTGTAG	145
*GAPDH*	CTG CAC CAC CAA CTG CTT AG	AGC TCA GGG ATG ACC TTG C	219

## Results

### Fabrication and characterization of electrospun PCL nanofibrous scaffold

**Figure 1:**
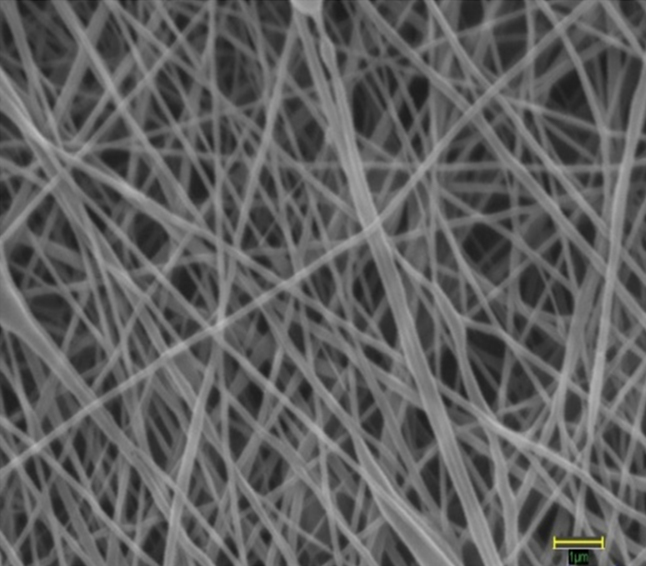
Architecture of electrospun PCL nanofiber scaffold as seen under a scanning electron microscope at 25,000× magnification. The average fiber diameter of nanofibers was 132±42 nm. Scale bar measures 1 µm.

**Figure 2:**
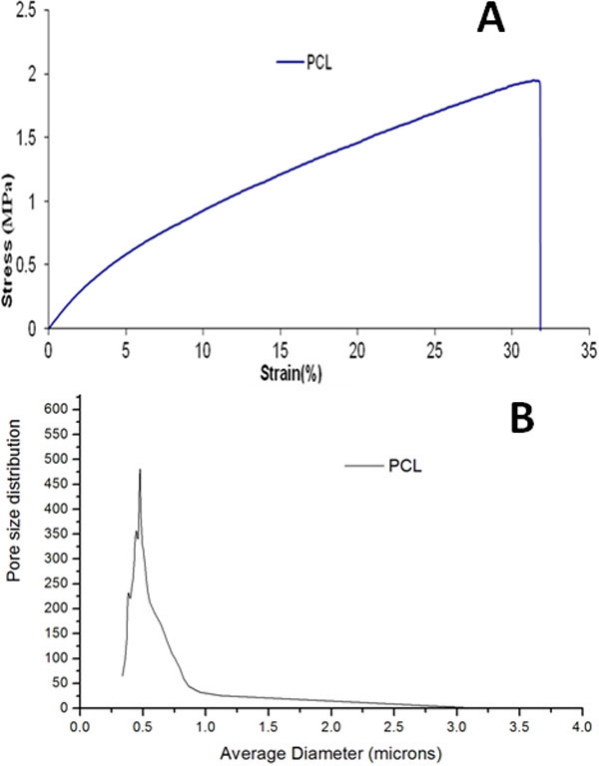
Physical characterization of electrospun PCL nanofibers. **A**: Tensile stress-strain curve of electrospun PCL nanofiber membranes showing tensile strength of 1.74±0.18 MPa and breaking strain of 30.08±2.66%. **B**: The graph shows pore size distribution of nanofiber membranes in the range of 0.2–4 microns.

The SEM images showed high interconnectivity of pores with random deposition of nanofibers (Figure 1). The average fiber diameter was found to be 132±42 nm, with a water contact angle of 90⁰. Figure 2A shows a typical stress-strain curve obtained for PCL nanofibrous scaffolds. The average tensile strength of PCL scaffolds was 1.74±0.18 MPa (Mega Pascal); breaking strain was 30.08±2.66%. The pore size of the nanofibrous membranes varied in the range of 0.2–4.0 microns (Figure 2B). The scaffolds were found to be highly porous, with average porosity of 85% (Table 2).

**Table 2:** Physical properties of electrospun PCL nanofibrous scaffolds.

**Property**	**PCL scaffold**
Water contact angle (degree)	90
Tensile strength (MPa)	1.74±0.18
Breaking strain (%)	30.08±2.66
Pore size (microns)	0.2–4.0
Porosity (%)	85
Fiber Diameter (nm)	132±42

**Figure 3:**
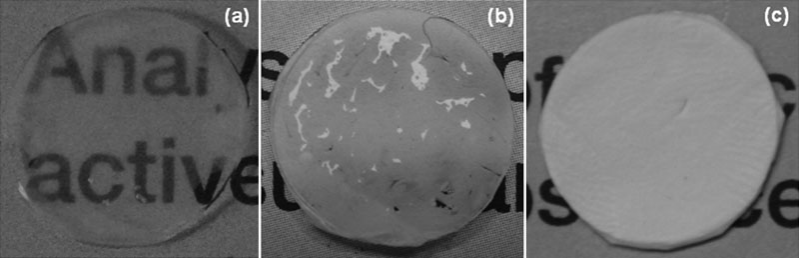
Optical transparency of PCL nanofiber membranes and HAM. **A**: Wet HAM, showing transparency through which the printed text can be easily read. **B**: Wet PCL membrane showing translucency through which the printed text is slightly visible. **C**: Dry PCL membrane showing complete opacity and the text underneath can not be read through it.

PCL membrane dipped for 48 h in PBS showed better wetting than samples dipped for 2 h or 24 h. Therefore, PCL samples dipped for 48 h were used for assessment of transparency. Figure (3A-C) shows a digital photograph for visual assessment of transparency of wet HAM as well as wet PCL and dry PCL membranes, all supported on a glass coverslip. Dry PCL membrane is completely opaque and the text underneath can not be read through it. In contrast, the wet PCL (48 h dipped) membrane shows translucency, as the printed text underneath is slightly visible. However, the transparency shown by wet PCL membrane is less than that of wet amniotic membrane, through which the text can be easily read.

**Figure 4:**
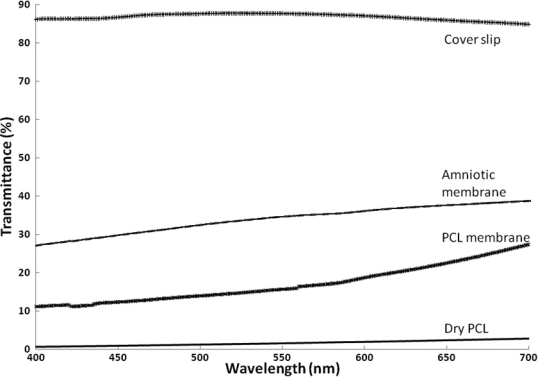
Optical transmittance of PCL nanofiber membranes, HAM and glass coverslips. Glass coverslips (control) showing maximum transmittance of 85% at a wavelength of 700 nm. HAM showing 38% of transmittance at a wavelength of 700 nm, whereas wet PCL membrane showing 27% transmittance and dry PCL membrane showing only 3% light transmittance at a wavelength of 700 nm.

The transparency of the three samples was also compared quantitatively using UV-Visible spectroscopy. From the UV-Vis spectra (Figure 4), it can be observed that glass coverslips (taken as control) showed maximum transmittance of about 85%. In contrast, the opaque dry PCL membrane showed less than 3% of light transmittance throughout the wavelength range. However, wet PCL membrane showed a significantly higher transparency, with about 28% transmittance at the wavelength of 700 nm and 11% at the wavelength of 400 nm. These values are only slightly lower than those for amniotic membrane, which showed transmittance of 38% and 27% at 700 nm and 400 nm, respectively. Hence, it may be inferred that PCL membranes in a wet state have significantly higher transparency when compared with dry PCL membrane. Yet, its transparency is lower than that of the wet amniotic membrane, which is considered as a standard.

### Biocompatibility assessment of nanofibers

**Figure 5:**
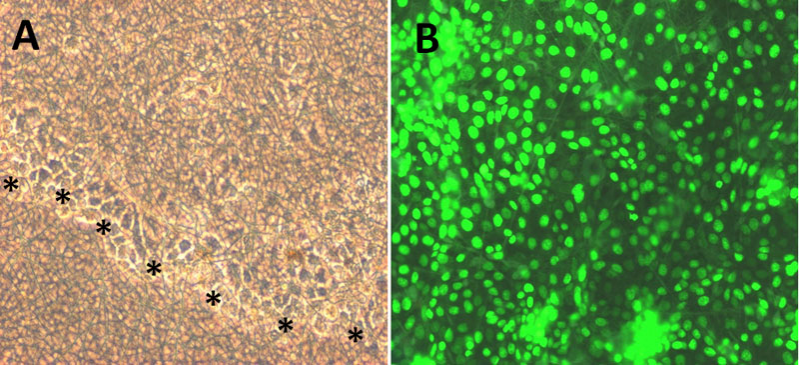
Biocompatibility assessment of electrospun PCL nanofibers: **A**: Phase contrast pictures shows migration of HCE-T cells over nanofibers (black stars line). **B**: Epithelial cell sheet demonstrates high viability ratio of HCE-T cells on nanofibers by their positive green staining. Cells were observed at 200× magnification.

HCE-T cells showed formation of a continuous epithelial cell sheet as observed through a phase contrast microscope (Figure 5A). Viability staining depicted the presence of live and dead cells. Live dye is a cell permeable dye that gives green fluorescence, whereas PI is a non-permeable dye that stains dead cells and gives red fluorescence. After seven days, HCE-T cells cultured over nanofibers showed 100% cell viability without any signs of cell death, proving that the scaffold is biocompatible and did not hamper cells from growing over the scaffold (Figure 5B).

**Figure 6:**
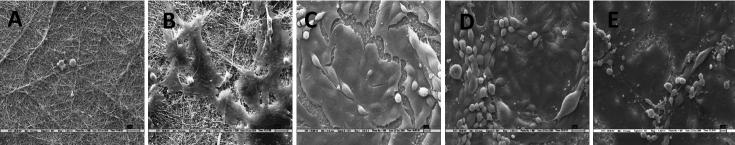
SEM of HCE-T cells seeded over nanofibers to evaluate the attachment ability at different time intervals. **A**: SEM after 6 h post seeding shows almost no cell attachment. **B**: After 1st day, cells attached to the polymer surface, become large and flat in morphology. **C**: After 3rd day, SEM revealed confluent monolayer formation over nanofibers with good cell spreading. **D**: Micrograph on the 5th day depicted good cell attachment and spreading on the nanofibers surface. **E**: Micrograph on day 7 illustrated confluent epithelial layer over nanofibers surface similar as day five.

SEM images showed very few or almost no cell attachment after 6 h post-seeding (Figure 6A). However, after 24 h of culture, cells were able to attach to the polymer surface (Figure 6B), and gradually increase in number over time. The day 3 image demonstrated the formation of a monolayer from the periphery to the center of polymer, whereas on day 5, cells started becoming multi-layered (Figure 6C,D). Day 7 images (Figure 6E) indicated almost equal distribution of cells, the same as on day 5 (Figure 6D). Cells were successfully adhered and were able to form a healthy, thick cellular layer over the polymer surface without any morphological disorder. The cell attachment continued to increase over time. The results indicated that polymer is biocompatible and does not invoke any cytotoxic effects to the HCE-T cell. This finding is consistent with the idea that nanofibers may have the potential to support the LECs’ attachment and proliferation in-vitro.

### Cellular response of LECs cultivated over nanofibers

#### Morphological observation

**Figure 7:**
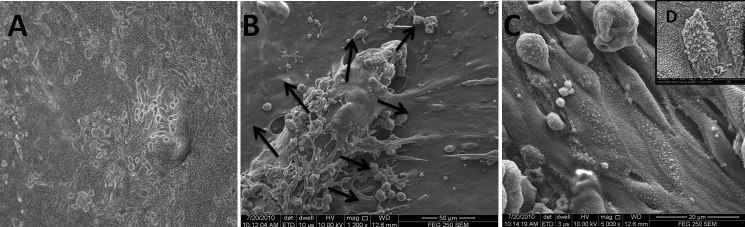
Culture of limbal epithelial cells cultivated on electrospun PCL nanofibers by explant method. **A**: Confluent limbal epithelial cell sheet grown on PCL nanofibers with round and ovoid morphology examined using phase contrast microscopy at day 14 (100× magnification). **B**: SEM image, LECs’ growth initiated from the edge of the explant (black arrowhead). **C**: SEM image showing that epithelial cells are closely attached to each other with tightly opposed cell junctions, and **D**: apical surface showing numerous short microvilli.

Limbal epithelial cells migrated from the periphery of the explant within 1–2 days, and further formed a monolayer within 7–10 days. After 10–12 days, the formation of a healthy and densely populated epithelial cell sheet was seen (Figure 7A,B). SEM images (Figure 7C) depicted a continuous layer of flat, hexagonal epithelial cells with a cobblestone-like appearance and firm attachment to the polymer surface. The apical surface of the cells was covered with numerous short microvilli that were closely attached to each other. Distinct cell boundaries and tightly opposed cell junctions were also evident (Figure 7D).

#### Cellular viability and proliferation

**Figure 8:**
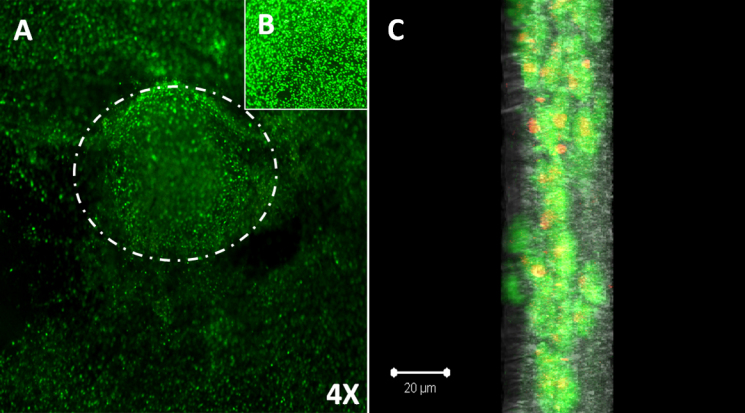
Viability Staining of LECs cultivated over PCL nanofibers. **A**: LECs depicted high ratio of viable cells as demonstrated by positive green staining. Phase contrast micrograph shows that LECs are migrating from the periphery of viable limbal explant (white arrowhead; 40× magnification). **B**: LECs cultivated on electrospun nanofibers shows confluent viable cell sheet at 100× magnification. **C**: Confocal microscopy depicted LESCs infiltrated the nanofibers and formed viable 3D corneal epithelium, positive viability staining (green) nanofibers (gray).

**Figure 9:**
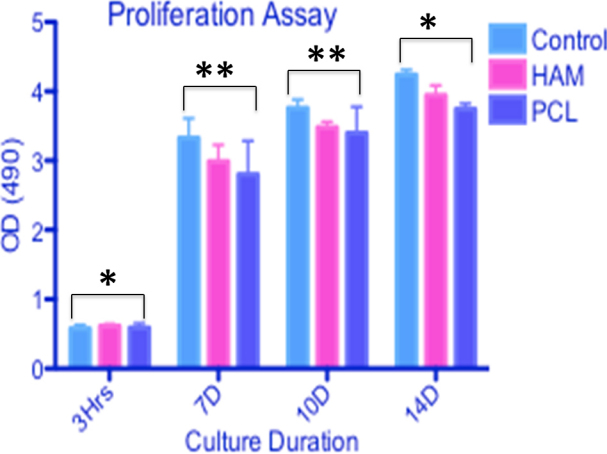
Cell proliferation (MTT) assay of LECs. LECs cultivated on electrospun nanofiber scaffold and HAM at different time intervals (days 0, 7, 10, and 14) and their proliferation potential compared with glass coverslips (control). Data represents three independent experiment and all data points plotted as mean values±SD (*p<0.001, **p<0.05).

LECs cultured for two weeks on nanofibers showed a 96% viability ratio, as demonstrated by green and red staining (Figure 8A,B). Confocal microscopy illustrated that cells infiltrated the nanofibers and remained viable there for two weeks, as confirmed by positive viability staining. Micrograph showed the formation of a three-dimensional (3D) construct (Figure 8C).The MTT data (Figure 9) also indicated that LECs remain viable on polymer surfaces for up to two weeks similar to HAM and control, and continued to increase in number over time. The proliferative capacity of LECs was less on nanofibers as compared to cells cultivated on HAM and glass coverslips (p<0.05). LECs showed almost equivalent metabolic activity on day 1 over HAM and glass coverslips, but thereafter, their metabolic activity increased on the control and HAM surface as compared to nanofibers.

#### Molecular characterization

**Figure 10:**
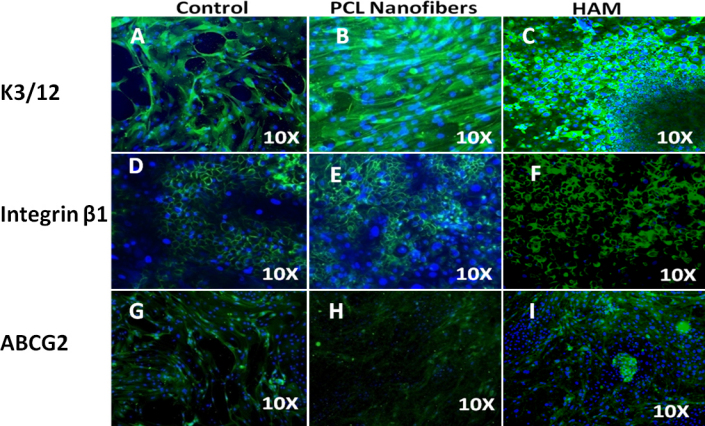
Expression of stem/progenitor cells (integrin β1, ABCG2) and differentiation-associated markers (K3/12) in ex vivo–expanded human LECs. Immunofluorescence staining shows positive expression of cytokeratin; (**B**), K3/12, (**E**) integrin β1, (**H**) ABCG2 on PCL nanofibers similar to coverslip (control; **A**) K3/12, (**D**) integrin β1, (**G**) ABCG2 and HAM (**H**) K3/12 (**F**), integrin β1, (**I**) ABCG2.

**Figure 11:**
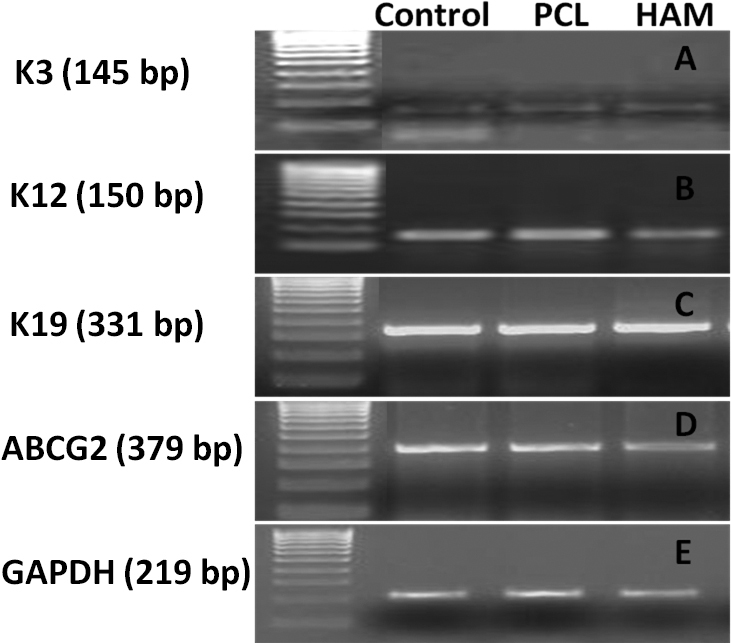
RT PCR analysis of LECs expanded over PCL nanofibers, HAM and control surface (coverslip): Differentiation markers (**A**) *K3* (145 bp), (**B**) *K12* (150 bp) and stem cell–associated (**C**) *K19* (331 bp), (**D**) *ABCG2* (379 bp), (**E**) *GAPDH* (219 bp) used as an internal control.

The phenotype of LECs was examined using immunofluorescence (K3/12, Integrin β1 and ABCG2) and RT PCR (K3, K12, K19, and ABCG2). Figure 10A-C showed bright positive staining for the cornea-specific cytokeratin K3/12. LECs cultivated over nanofibers showed positive immunostaining for integrin β1, similar to the control and HAM (Figure 10D-F). Moreover, the ABCG2 putative stem cell marker (Figure 10G-I) was also noticed in a few cells. These findings were further validated by RT PCR, and the results confirmed the expression of stem cell markers (ABCG2 and K19) as well as differentiation markers (K12 and K3; Figure 11).

## Discussion

In recent years, many carriers have been used to culture LECs; however, the majority of previous studies have used HAM as a choice of carrier due to its low-cost procurement, easy availability, and ability to maintain stemness of the culture, promote epithelialization, and reduce inflammation and scarring [11-13,28-33]. Nonetheless, due to its biologic origin, HAM carries inherent risks such as disease transmission and infection that cannot be totally avoided [14,15]. The use of synthetic biomaterial can eliminate the risk factors associated with biologic materials. Various natural and synthetic ECMs have been used previously for ocular surface bioengineering [34] such as fibrin [6,7], collagen [8,9], HAM [1-4], temperature responsive cell culture surfaces [5,17], human anterior capsule [35], chitosan [36,37], Mebiol gel [38], and silicon [10]. Here, our aim is to develop a biocompatible synthetic stromal substrate that can work as an excellent scaffold for LEC expansion and should share a close resemblance to the native ECM.

PCL consists of synthetic aliphatic polyesters that are bioresorbable and biocompatible. Extensive research has been conducted on PCL’s biocompatibility and efficacy, in vitro and in vivo. PCL is an FDA-approved material for several medical applications such as drug delivery devices, sutures, and adhesion barrier [24,39]. PCL is being extensively investigated as a scaffold for tissue repair in tissue engineering. In ophthalmic applications, PCL has already been explored as a drug delivery agent and as a carrier to cultivate retinal and conjunctival progenitor cells [21-23] due to its in vivo biocompatibility. It does not induce any immunological reactions after degradation. One disadvantage of PCL is its hydrophobicity. However, its surface properties can be easily altered using surface modification techniques or by blending PCL with other polymers with desired functional groups to make them more effective as biomaterials. Further investigation is underway for improving PCL’s biocompatibility and optical transparency. It is one of the most highly exploited synthetic polymers in the field of tissue engineering owing to its various advantages [39-44]. The SEM, porometer, and mechanical strength data of PCL scaffolds show that it possesses a high surface-to-volume ratio, high porosity, and sufficient tensile strength. These findings indicate that the fabricated electrospun PCL nanofibers possess the required properties and can provide optimum fluid and nutrient transport, bio-absorbability, mechanical integrity, and an ideal geometry for the construction of desired ocular surfaces.

Studies on biocompatibility were performed using an HCE-T cell line because the corneal epithelial cell line closely mimics primary LEC culture in terms of morphology and expression profile (unpublished data). This cell line was used for initial standardization of experiments because it diminished variation between the experiments. This variation was due to biologic variability between donor tissues and the limited availability of human tissue. Using this approach, we tried to address the question of cell survival, attachment, and proliferation. SEM results provided information regarding the cell-substrate interaction, which is a critical parameter for successful cell growth. HCE-T cells were able to form a healthy and continuous epithelial sheet over nanofibers, which were firmly attached to the polymer surface and were able to retain their viability and native phenotype. Epithelial cells are anchorage-dependent; their survival and proliferation ability can also be affected by cell-surface interaction. The biocompatibility results indicate that fabricated nanofibers were able to support cell attachment, proliferation, and the viability of the cultured cells. The biocompatibility studies demonstrated several encouraging findings, on the basis of which PCL scaffolds were further used for LEC expansion.

In this study, we have evaluated the adhesion, growth, motility, and phenotype of the LECs cultured on electrospun PCL nanofibers. SEM findings substantiate the hypothesis that the structural properties and architecture of the randomly deposited electrospun nanofibers are able to create natural ECM for cell growth and have an effect on cellular attachment and proliferation. High cell viability suggests that under our fabrication conditions, PCL nanofibers could provide favorable growth conditions for cell survival and offer optimum nutrient and gas exchange for cell growth, even when cells have penetrated the scaffolds. PCL is also helpful in providing sufficient gas and nutrient exchange required for quick wound healing on ocular surfaces. Confocal microscopy results suggest the formation of a functional 3D corneal epithelium as cells proliferate on the surface, infiltrate the scaffold, and remain viable for two weeks. This finding substantiates the idea that the pore size of the nanofiber webs was optimum for cells to drift inside the polymer web, and the nanofiber scaffold may closely biomimic a natural ECM. 3D architecture of the scaffold increases the cell packing capacity, which in turn increases the load of stem cells inside the scaffold for Limbal Stem Cell Transplantation (LSCT) as compared to conventional LSCT using HAM as a scaffold. The latter method allows proliferation on only one side of the scaffold.

Viability staining and MTT results of the LECs demonstrated that cells display a high survival rate over a period of two weeks in in vitro culture conditions, which is the required optimum time for confluent epithelial cell sheet formation. This supports the idea that PCL is a non-toxic and biocompatible material. MTT assay showed that cells cultured on a PCL surface were metabolically active (p<0.05); however, their activity was less compared with cells cultured over glass coverslips. This may be due to the hydrophobic nature of PCL, which undermines proper cellular adherence. Overcoming PCL’s hydrophobicity is one of the issues that is under further investigation.

The intensity of purple-colored formazan crystals increased with time, indicating that cellular proliferation was enhanced. This implied that the scaffolds were suitable for supporting cell growth. IF and RT PCR results suggested that the polymer surface did not lead to any change in the phenotypic features of LECs, since the molecular expression was the same as for the control surfaces. ABCG2 has been proposed as a universal marker of stem cells [45]. de Paiva et al. [46] have immunolocalized the expression of ABCG2 in the cell membrane and cytoplasm of the human limbal basal epithelial cells, but not in most limbal suprabasal cells and corneal epithelial cells. The ABCG2 expression suggests that the PCL surface was helping to maintain the “stemness’’ of the culture and did not induce complete differentiation. Integrin β1 and K19 have been identified as epithelial stem cell markers [27,47,48]. These results suggest that cultures were able to maintain their epithelial stem cell properties. K3 and CK12 are markers for a mature corneal epithelium and are absent from the basal layers of the limbal epithelium [49]. Expression of K3/12 suggests that cultivated limbal epithelial stem cells have the potential to differentiate into mature corneal epithelial cells. The expression of differentiation markers (K3 and K12) as well as stem cells’ associated markers (ABCG2, K19, and β1 Integrin) revealed that the LEC culture contained a heterogeneous cell population of differentiated as well as progenitor cells.

Two important observations were noticed in this study. First, 3D porous nanofibers were helpful in the formation of a functional three-dimensional corneal epithelial structure, which in turn facilitated the development of a normal corneal phenotype and the retention of the progenitor stem cell marker expression. Second, the scaffold surface supported the formation of a 3D corneal epithelium. This was true even with small pieces of tissue biopsy, which is advantageous for cell culture-based therapy. Collectively, all the data indicate that PCL nanofibers are suitable candidate for LEC cultivation, as they are non-toxic, have sufficient mechanical strength and optimum pore size, promote cell proliferation, and maintain the ‘‘stemness’’ of the culture.

### 

#### Conclusion

Our study showed that the PCL nanofibers have the potential to support the attachment and expansion of LECs without altering the phenotype, and can be further used to restore damaged ocular surfaces. Based on in vitro analysis, we hypothesized that PCL nanofibrous scaffold would provide a means to transplant confluent, stratified limbal epithelial cell sheet onto a limbal stem cell-deficit eye and would thus serve as an excellent candidate for ocular surface engineering.
